# Persistence with dimethyl fumarate in relapsing-remitting multiple sclerosis: a population-based cohort study

**DOI:** 10.1007/s00228-017-2366-4

**Published:** 2017-11-11

**Authors:** Irene Eriksson, Thomas Cars, Fredrik Piehl, Rickard E. Malmström, Björn Wettermark, Mia von Euler

**Affiliations:** 1 Department of Medicine Solna, Karolinska Institutet, Stockholm, Sweden; 2 Department of Healthcare Development, Stockholm County Council, Stockholm, Sweden; 3 Department of Medical Sciences, Uppsala University, Uppsala, Sweden; 4 Department of Clinical Neuroscience, Karolinska Institutet, Stockholm, Sweden; 5 Clinical Pharmacology, Karolinska University Hospital, Stockholm, Sweden; 6 Department of Clinical Science and Education, Södersjukhuset, Karolinska Institutet, Stockholm, Sweden

**Keywords:** Multiple sclerosis, relapsing-remitting, Dimethyl fumarate, Drug utilization, Persistence

## Abstract

**Purpose:**

To describe patients initiating dimethyl fumarate (DMF) and measure persistence with DMF, discontinuation, and switching in treatment-naïve DMF patients and patients switching to DMF from other multiple sclerosis disease-modifying treatments (DMTs).

**Methods:**

A population-based cohort study of all Stockholm County residents initiating DMF from 9 May 2014 until 31 May 2017. All data were derived from a regional database that collects individual-level data on healthcare and drug utilization of all residents. The study outcomes were persistence with DMF and DMF discontinuation and switching to other DMTs. Persistence was measured as the number of days until either DMF discontinuation (treatment gap ≥ 60 days) or switching to another DMT.

**Results:**

The study included 400 patients (median follow-up = 2.5 years). The majority had previously been treated with other DMTs (61%). Throughout the follow-up period, 124 patients (31%) discontinued DMF and 114 patients (29%) switched treatment. Overall, 34% of patients initiating DMF stopped treatment within 1 year and only 43% of patients remained on DMF at 2 years from treatment initiation.

**Conclusions:**

DMF had a rapid market uptake likely due to high expectations held by both patients and clinicians. However, persistence with DMF in routine clinical practice was found to be low.

**Electronic supplementary material:**

The online version of this article (10.1007/s00228-017-2366-4) contains supplementary material, which is available to authorized users.

## Introduction

The introduction of interferon therapy improved the prognosis of relapsing-remitting multiple sclerosis (RRMS) [1]. However, a significant unmet medical need has remained for patients with treatment intolerance and for those who experience disease progression while on therapy. Consequently, anticipation and expectations for the next generation disease-modifying treatments (DMTs), particularly oral formulations, have been high [2]. The first oral DMT for RRMS patients, fingolimod, was introduced in 2011 in Europe [3] followed by teriflunomide and dimethyl fumarate (DMF) in 2013 [4, 5]. There have also been new hospital-administered DMTs developed, including alemtuzumab and daclizumab [6]. In addition, in recent years, the B-lymphocyte-depleting drug rituximab has increasingly been used off-label for treatment of both RRMS and, to some extent, progressive MS, especially in Sweden [7, 8].

The rapidly changing MS treatment landscape poses a challenge for clinicians to select the most optimal DMT for their patients. Even though risk-benefit profiles of the new DMTs have been assessed in clinical trials, there are uncertainties about their effectiveness and safety as well as long-term outcomes when used in routine clinical practice [9, 10].

When DMF was introduced, the expectations were high both from patients and clinicians [2]. On the other hand, there were concerns about increasing costs as DMF is more expensive than existing injectable DMTs [11, 12] and about dropout rates that were relatively high in the pivotal DMF trials [13, 14]. However, so far, real-world data on DMF are scarce. In this study, we analyzed DMF utilization patterns in routine clinical practice using a database that contains individual-level data on healthcare and drug utilization of all residents of the largest administrative region in Sweden.

## Methods

### Study design

This is a population-based cohort study of all Stockholm County residents initiating DMF (Tecfidera) from 9 May 2014 (the date when reimbursement of DMF was approved in Sweden) until 31 May 2017. The study was approved by the regional ethics committee in Stockholm, Sweden (Ref. no. 2015-2329-31-4).

### Data sources

All data used in our analyses were derived from VAL, an in-house regional repository that collects reimbursement claims and other health-related data of all Stockholm County residents (2.3 million; approximately 23% of the population of Sweden) [15–17].

We used hospital discharge (inpatient) data and data on outpatient specialist and primary care visits to obtain information on diagnoses [International Classification of Diseases (ICD)-10], procedures [Swedish Classification of Health Interventions and the Nordic Medico-Statistical Committee (NOMESCO) codes] [18] as well as overall utilization of healthcare services from 1 January 2010 until 31 May 2017.

Information on outpatient drug utilization was derived from outpatient pharmacy dispensation data [19]. DMTs administered in hospitals were identified using procedure codes and drug codes [Anatomical Therapeutic Chemical (ATC) classification] recorded in the inpatient and outpatient specialist visit data. Data on drug utilization were derived from 1 July 2010 until 31 May 2017.

Information on patient age, sex as well as migration and death records were also obtained from VAL.

### Study population

We selected all Stockholm County residents initiating DMF from 9 May 2014 until 31 May 2017. The index date was defined as the date of the first DMF dispensation. In this population, we then identified MS treatment-naïve DMF patients and patients switching to DMF from other DMTs. Treatment-naïve DMF patients were defined as patients who had no history of dispensation or inpatient administration of DMTs for at least 1 year prior to the index date. To allow for assessment of baseline characteristics and prior DMT use, patients with less than 1 year of continuous residence in the Stockholm County prior to the index date were excluded.

Patients were followed from the index date until the earliest of the following: the end of continuous residence in the Stockholm County or the end of study period (31 May 2017). Furthermore, because women are advised to discontinue DMTs during pregnancy, women with a delivery diagnosis code (ICD-10 codes: O80-O84) were censored 280 days prior to the delivery date.

### Study variables

#### Exposure

The following DMTs were available in the Stockholm County during the study period: interferon-beta-1a, peginterferon-beta-1a, interferon-beta-1b, glatiramer acetate, fingolimod, dimethyl fumarate, teriflunomide, natalizumab, alemtuzumab, daclizumab, and rituximab though not formally approved as an MS DMT. ATC codes were used to identify the study drugs in the database (see Online Resource 1).

#### Outcomes

The primary study outcome was persistence with DMF. We also assessed DMF discontinuation and switching to other DMTs. To operationalize the outcome definitions, we first identified treatment gaps. Treatment gap was measured as time between the end of DMF supply dispensed (dispensation date plus days of supply) until the date of the next dispensation. Days of DMF supply were estimated using the information on type and number of dispensed packages and typical DMF administration regimen (twice daily). The estimated days of supply were then used in the calculation of the variables related to treatment gaps.

We assumed that patient discontinued DMF if they had a treatment gap of at least 60 days. Time to discontinuation (in days) was measured as time between the index date and the end of supply of the DMF prescriptions dispensed. If after this gap the patient was dispensed DMF, we classified this as a restart of DMF treatment. Information on the number of patients restarting DMF was provided for descriptive purposes.

Patients with a dispensation or in-hospital administration of another DMT within the gap period were classified as switching. Time to switching (in days) was measured as time between the index date and the dispensation or in-hospital administration date of the subsequent DMT. We calculated time to switching only for the first switching. In addition, we described which other DMTs DMF patients switched to regardless of the length of the gap period.

Persistence was measured as the number of days from the index date until either discontinuation or switching to another DMT (if switching occurred after the end of DMF supply, we used the number of days from the index date until the end of DMF supply).

We also described prior DMT utilization patterns in patients switching to DMF from other DMTs (with data available as far back as 1 July 2010).

### Covariates

We obtained baseline patient information on age, sex, and history of relapses. We also identified commonly reported comorbidities and comedications during 1 year prior to the index date (see Online Resource 2 for the ICD-10 and ATC codes used). Healthcare resource utilization at baseline during 1 year prior to the index date was measured as the number of hospitalizations and in-hospital days, number of outpatient specialist visits, number of primary care visits, and number of drug classes (as defined by ATC classification level 4) [20] dispensed.

Based on an adaptation of an algorithm developed for US claim data [21], we defined as a relapse: (1) hospital admission in neurology, internal medicine, or pediatrics departments with a diagnosis of MS (ICD-10 code: G35) and/or (2) outpatient specialist visit with a diagnosis of MS (ICD-10 code: G35) as a primary diagnosis with either an administration of glucocorticoids (dexamethasone, ATC code H02AB02, or methylprednisolone, ATC code H02AB04) in the outpatient specialist clinic or with a dispensation of prednisone (ATC code H02AB07) from an outpatient pharmacy within a week following the visit.

### Statistical analyses

Descriptive statistics were used to summarize data on baseline characteristics of the study population. For categorical variables, we reported frequencies and proportions. For continuous variables, we calculated the mean, standard deviation (SD), median, and range.

Persistence was plotted using Kaplan-Meier survival curves. In our sensitivity analyses, we changed the permissible treatment gap period to 30 and 180 days.

Data management and analyses were conducted using SAS 9.4 (Cary, NC).

## Results

**Fig. 1:**
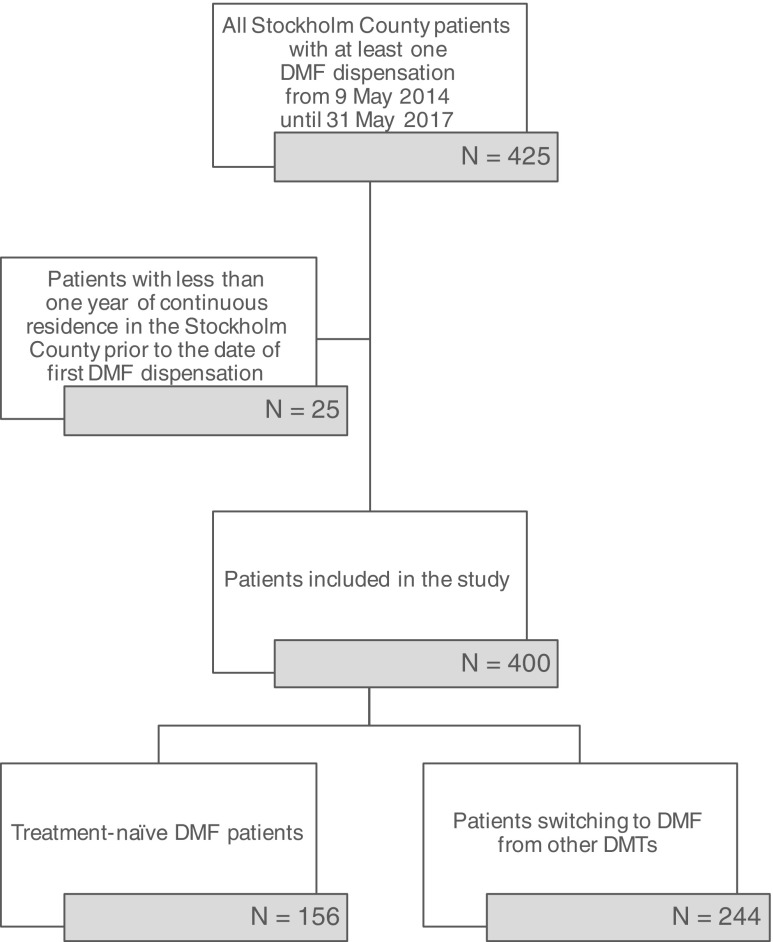
Cohort selection flowchart

During the study period, 425 patients initiated DMF in the Stockholm County; of these, 400 met our study’s selection criteria (see Fig. 1).

**Fig. 2:**
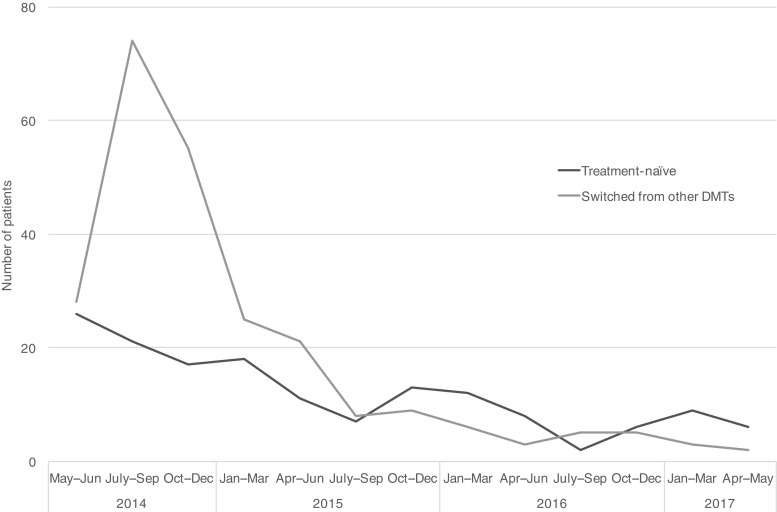
DMF initiation patterns from May 2014 to May 2017

One hundred and fifty-six patients (39%) were treatment-naïve and 244 (61%) were previously treated with other DMTs. From May to December 2014, 221 patients initiated DMF with the majority (71%) switching to DMF from other DMTs. There were fewer patients starting each subsequent year: 112 in 2015 and 47 in 2016. The initiation patterns are illustrated in Fig. 2 and baseline characteristics of the study patients are presented in Table 1 (for information on comorbidities, comedications, and healthcare resource utilization see Online Resource 3). Patients switching to DMF from other DMTs were older compared to the treatment-naïve (40.5 vs. 35.3 years old, respectively). Among the treatment-naïve DMF patients, 24% had experienced a relapse in the year prior to treatment initiation; the corresponding proportion for patients switching from other DMTs was 10%.

**Table 1:** Baseline characteristics of patients initiating DMF

	Treatment-naïve DMF patients	Patients switching to DMF from other DMTs
	*N* = 156	*N* = 244
Age (years), mean (SD)	35.3 (9.8)	40.5 (10.1)
Age groups, *N* (%)		
< 25	20 (13%)	14 (6%)
25–35	61 (39%)	52 (21%)
35–45	47 (30%)	91 (37%)
> 45	28 (18%)	87 (36%)
Female, *N* (%)	113 (72%)	186 (76%)
Relapse in the past year, *N* (%)	37 (24%)	24 (10%)

Of those 244 patients who switched to DMF from other DMTs, most switched from interferon-beta-1a, Avonex (101; 41%) and Rebif (48; 20%), followed by glatiramer acetate Copaxone (39; 16%), interferon-beta-1b Betaferon (20; 8%), and natalizumab Tysabri (17; 7%). Thirty-eight out of 244 switchers used more than one DMT prior to DMF with the majority of these patients treated with multiple injectable DMTs (interferon-betas and glatiramer acetate) prior to initiating DMF.

Descriptive analyses of switching patterns (without accounting for the length of the treatment gap) showed that within 1 year of DMF initiation, 80 patients (20%) switched to another DMT; most to rituximab (48 patients) (see Online Resource 4). When extending the follow-up period to 2 years, the number of patients switching to another DMT increased to 134 (34%) with the majority switching to rituximab (89 patients). When including all available follow-up data (mean follow-up = 789 days [SD = 297], median = 910, min = 15, max = 1109), 153 DMF patients (38%) switched. Rituximab was by far the most common DMT that patients switched to during the follow-up period (see Online Resource 4).

Using a treatment gap of 60 days, we identified 124 patients (31%) discontinuing their treatment within the entire follow-up period. The mean time to discontinuation was 366 days (ranging from 7 to 1073 days). Twenty-seven of these 124 patients (22%) would later restart DMF treatment (in the persistence analyses, these 27 patients were considered non-persistent from the beginning of the first 60-day gap period encountered in the data). One hundred fourteen patients (29%) were classified as switching treatment. The mean time to switching was 372 days (ranging from 7 to 958 days).

**Fig. 3:**
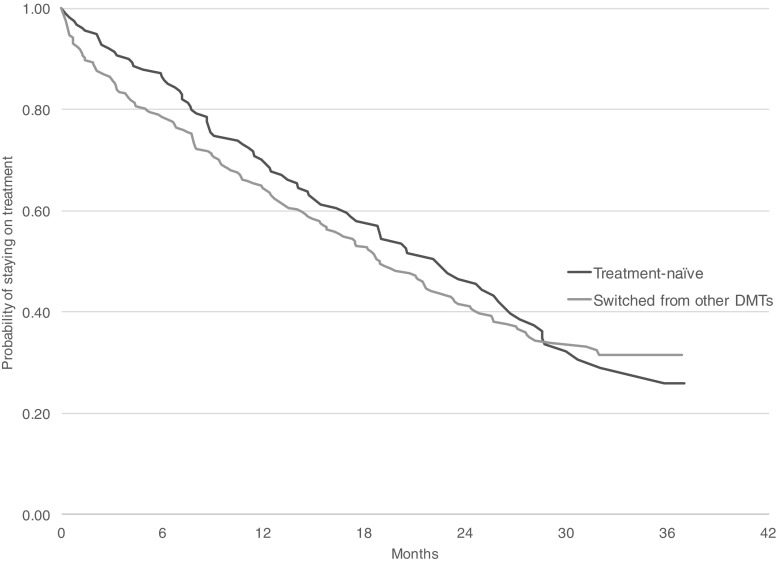
Persistence with DMF in treatment-naïve DMF patients and in patients switching to DMF from other DMTs

Overall, 34% of patients stopped DMF treatment within 1 year. After 2 years of follow-up, only 43% of patients remained on DMF (see Online Resource 5). Among treatment-naïve patients, 69 and 46% were persistent with DMF at 1 and 2 years, respectively. The corresponding numbers for patients who switched to DMF from other DMTs were 64 and 40%, respectively (see Fig. 3). There were numerical differences in persistence between women and men (62 and 40% of women and 78 and 51% of men were persistent at 1 and 2 years, respectively).

The results of sensitivity analyses varied according to the treatment gap chosen but were generally in line with the analyses based on the 60-day gap period (see Online Resource 6). Using a 30-day gap, persistence with DMF at 2 years in treatment-naïve and patients switching to DMF was 38 and 35%, respectively. The 180-day gap correspondingly provided the higher estimates (48 and 46% at 2 years in treatment-naïve and patients switching to DMF, respectively).

## Discussion

The results of this population-based observational study indicate that persistence with DMF in routine clinical practice is low. One third of patients initiating DMF stopped treatment within 1 year of follow-up, and at 2 years only 43% of patients remained persistent. DMF was the first oral first-line DMT introduced, and the initial market uptake was relatively rapid compared to that of other recently introduced DMTs, suggesting that the expectations for DMF were high at the time of introduction. The majority of patients initiating DMF were switching from other DMTs, most frequently interferon-betas, but the persistence was poor regardless whether or not patients had prior experience of DMT. A switch to DMF could have been driven by a number of factors. Treatment failure on previous treatment was likely not a major driving factor as only 10% of the switching patients had data indicating a recent relapse (in our data we however could not identify patients showing signs of neuroradiological progression as evidence of insufficient treatment effect). Still, patient preference for an oral administration regimen is a more likely explanation in most cases. The peak in uptake happened soon after DMF reimbursement, driven by DMT-experienced patients, indicating that patients and clinicians were awaiting DMF and switched as soon as it became available.

After stopping DMF, most of the patients switched to rituximab. The effectiveness and safety of rituximab in RRMS has been extensively studied in Sweden [8, 22–25]. During the time period reported in our study, an increasing use of rituximab off-label for MS occurred in the Stockholm County. It is difficult to calculate the impact of this on the retention rate of DMF. However, it is reasonable to assume that a perceived better tolerability profile of rituximab led to a decreased threshold to switch from DMF to rituximab compared to a situation in which the rituximab option had been lacking.

DMF was approved based on two randomized clinical trials: Determination of the Efficacy and Safety of Oral Fumarate in Relapsing-Remitting MS [DEFINE] [13] and Comparator and an Oral Fumarate in Relapsing-Remitting Multiple Sclerosis [CONFIRM] [14]. In comparison with the trials, our DMF population had more patients who had been previously treated with other DMTs (61 vs 40% in DEFINE and 29% in CONFIRM). The mean duration of follow-up in our study was determined by the time when our data were analyzed, i.e., 3 years after DMF was introduced to the Swedish market (112.7 vs 83.9 weeks in DEFINE and 84.4 weeks in CONFIRM). A higher proportion of patients discontinued DMF in our study than what was seen in the trials (57 vs 31% in DEFINE and 30% in CONFIRM). As reported in DEFINE and CONFIRM, 16 and 12%, respectively, discontinued DMF due to adverse events. We were not able to assert the reasons for discontinuation as our analyses were based on administrative data but it is likely that lack of effectiveness, lack of tolerability, or a combination of the two contributed to the low persistence rates observed in our study.

Our findings of low DMF persistence are in line with the results of other observational studies. To date, studies out of the USA provide the majority of published data on DMF persistence in routine clinical practice. These studies were based on a variety of data sources, including reimbursement claims data [26–28], patient health records from a single or multiple clinics [29–31], and patient registries [32]. The follow-up period in these studies also varied from as short as 6 months to 2 years. The claims-based studies reported persistence ranging from 56 to 68% by the end of a 1-year follow-up [27, 28]. The findings of studies based on patient health records and patient registries were also similar showing that a considerable number of patients discontinued DMF [29–32]. One of these studies found that gastrointestinal adverse events were the most common reasons leading to discontinuation [31].

There are several strengths to our study. First, this is the first population-based study on DMF persistence in routine clinical practice. The data used in our study covered all residents from the largest administrative region in Sweden. Second, our data are routinely collected and include primary care, outpatient specialist visits, hospitalizations, and drugs used both in ambulatory care and in hospitals. In-hospital drug use (natalizumab, rituximab, and alemtuzumab) is documented using procedure codes which we previously validated using data from electronic health records [33]. Third, in our analyses, it was possible to follow up patients for as long as they lived in the region, enabling virtually a complete follow-up (of our study population, only eight patients emigrated from the region during the follow-up period).

The small number of patients included in our study is a limitation; however, the study still provides a large population compared to other observational studies to date [27, 29, 30, 32]. This small number, however, led to low power to detect factors associated with poor persistence. To avoid misclassification, we censored women stopping DMF treatment due to pregnancy based on a recorded delivery diagnosis code. However, this approach has a limitation as it does not sufficiently capture discontinuations among all women trying to conceive. Furthermore, as with any analysis based on administrative claims data, we did not have clinical information (e.g., magnetic resonance imaging findings or Expanded Disability Status Scale (EDSS)) that could explain reasons for DMF discontinuation or switching to another DMT. Moreover, the ICD-10 code used for MS does not allow differentiation between the types of MS (i.e., RRMS or progressive MS); however, in Sweden, DMF is only indicated for RRMS and it is likely that the vast majority of study patients had RRMS. Finally, as with any observational study based on secondary data, this study is dependent on the accuracy and completeness of the information recorded in the database. Drug dispensation data in administrative claim databases however have been shown to be of high quality [34–36]. Of note also is that MS care in our region is largely centralized to three university specialist clinics. The vast majority of DMF prescriptions was written in these clinics that have trained MS nurses available to support the patients with initiation of therapy. To optimize conditions for good tolerability, patients were instructed to prolong the duration of the twice daily 120 mg dose up to 4 weeks in case of side effects, to try a low dose of salicylic acid, and to take DMF with food.

In conclusion, DMF had a rapid market uptake likely due to high expectations held by both patients and clinicians. However, more patients discontinued DMF in routine clinical practice than in the pivotal trials. While our analyses could not discern whether this was due to lack of effectiveness or lack of tolerability, persistence nonetheless is an important indicator of how the drug performs in routine clinical practice.

## Electronic supplementary material

Online Resource 1 (PDF 14 kb)

Online Resource 2 (PDF 17 kb)

Online Resource 3 (PDF 108 kb)

Online Resource 4 (PDF 18 kb)

Online Resource 5 (PDF 20 kb)

Online Resource 6 (PDF 77 kb)
