# Supplementary material for: Persistence with dimethyl fumarate in relapsing-remitting multiple sclerosis: a population-based cohort study
Source: Eur J Clin Pharmacol. 2017 Nov 11;74(2):219–26. doi: 10.1007/s00228-017-2366-4 (PMC5765201; doi:10.1007/s00228-017-2366-4)
Supplement: Supplementary file 1 — (PDF 14 kb) [file 228_2017_2366_MOESM1_ESM.pdf]

**Online Resource 1. MS DMTs available in the Stockholm County during the study period**

| MS DMT | Brand name | ATC code | Data source |
|---|---|---|---|
| interferon-beta-1a | Avonex | L03AB07 | outpatient pharmacy dispensation data |
| | Rebif | L03AB07 | outpatient pharmacy dispensation data |
| peginterferon-beta-1a | Plegridy | L03AB13 | outpatient pharmacy dispensation data |
| interferon-beta-1b | Betaferon | L03AB08 | outpatient pharmacy dispensation data |
| | Extavia | L03AB08 | outpatient pharmacy dispensation data |
| glatiramer acetate | Copaxone | L03AX13 | outpatient pharmacy dispensation data |
| fingolimod | Gilenya | L04AA27 | outpatient pharmacy dispensation data |
| dimethyl fumarate | Tecfidera | N07XX09 | outpatient pharmacy dispensation data |
| teriflunomide | Aubagio | L04AA31 | outpatient pharmacy dispensation data |
| natalizumab | Tysabri | L04AA23 | inpatient and outpatient specialist visits data |
| alemtuzumab | Lemtrada | L04AA34 | inpatient and outpatient specialist visits data |
| daclizumab | Zinbryta | L04AC01 | outpatient pharmacy dispensation data |
| rituximab | MabThera | L01XC02 | inpatient and outpatient specialist visits data |
