# Supplementary material for: Persistence with dimethyl fumarate in relapsing-remitting multiple sclerosis: a population-based cohort study
Source: Eur J Clin Pharmacol. 2017 Nov 11;74(2):219–26. doi: 10.1007/s00228-017-2366-4 (PMC5765201; doi:10.1007/s00228-017-2366-4)
Supplement: Supplementary file 2 — (PDF 17 kb) [file 228_2017_2366_MOESM2_ESM.pdf]

Online Resource 2. Comorbidity definitions

Comorbidity	ICD-10 code	Setting	Position
Anxiety	F40, F41, F42, F43.1	Inpatient, outpatient specialist, primary care	Primary or secondary diagnosis
Depression	F32, F33, F34, F38, F39	Inpatient, outpatient specialist, primary care	Primary or secondary diagnosis
Hypertension	I10, I11, I12, I13, I15	Inpatient, outpatient specialist, primary care	Primary or secondary diagnosis
Hyperlipidemia	E78.0, E78.2, E78.4, E78.5	Inpatient, outpatient specialist, primary care	Primary or secondary diagnosis
Diabetes	E10, E11, E12, E13, E14	Inpatient, outpatient specialist, primary care	Primary or secondary diagnosis
Pain	G50.1, H57.1 ,H92.0, K10.2, K14.6, M25.5, M25.5B, M25.5C, M25.5D, M25.5E, M25.5F, M25.5G, M25.5H, M53.0, M53.1, M53.9, M54.2, M54.3, M54.4, M54.5, M54.6, M54.9, M77.4, M79.1, M79.1B, M79.1C, M79.1D, M79.1E, M79.1F, M79.1G, M79,1H. M79.2, M79.6, M79.6B, M79.6C, M79.6D, M79.6E, M79.6F, M79.6G, M79.6H, M79.9, R07.0, R07.1, R07.2, R07.3, R07.4, R09.1, R10.1, R10.2, R10.3, R10.4, R10.4X, R51.9, R52.9, H92.0, M53.0, M53.1, M54.2, M54.3, M54.4, M54.5, M54.6, M54.9P, M79.1, R07-P, R10.1, R10.3P, R10.4P, R51.-, R52.-	Inpatient, outpatient specialist, primary care	Primary or secondary diagnosis

Online Resource 2. Comedication definitions

Comedication	ATC code
Drugs for peptic ulcer and gastro-oesophageal reflux disease	A02B
Drugs for constipation	A06A
Vitamin A and D, incl. combinations of the two	A11C
Vitamin B12 and folic acid	B03B
Corticosteroids for systemic use, plain	H02A
Thyroid preparations	H03A
Tetracyclines, beta-lactam antibacterials, penicillins	J01A, J01C
Antiinflammatory and antirheumatic products, non-steroids	M01A
Opioids	N02A
Other analgesics and antipyretics	N02B
Antimigraine preparations	N02C
Antiepileptics	N03A
Anxiolytics	N05B
Hypnotics and sedatives	N05C
Antidepressants	N06A
