# Supplementary material for: Persistence with dimethyl fumarate in relapsing-remitting multiple sclerosis: a population-based cohort study
Source: Eur J Clin Pharmacol. 2017 Nov 11;74(2):219–26. doi: 10.1007/s00228-017-2366-4 (PMC5765201; doi:10.1007/s00228-017-2366-4)
Supplement: Supplementary file 3 — (PDF 108 kb) [file 228_2017_2366_MOESM3_ESM.pdf]

Online Resource 3. Baseline characteristics of patients initiating DMF – comorbidities

	**Treatment-naïve DMF patients N = 156**	**Patients switching to DMF from other DMTs N = 244**
N (%)		
Anxiety	6 (4%)	14 (6%)
Depression	5 (3%)	9 (4%)
Hypertension	1 (1%)	11 (5%)
Hyperlipidemia	0 (0%)	0 (0%)
Diabetes	1 (1%)	5 (2%)
Pain	49 (31%)	74 (30%)

Online Resource 3. Baseline characteristics of patients initiating DMF – comedications

	**Treatment-naïve DMF patients N = 156**	**Patients switching to DMF from other DMTs N = 244**
N (%)		
Drugs for peptic ulcer and gastro-oesophageal reflux disease	19 (12%)	44 (18%)
Vitamin A and D, including combinations of the two	13 (8%)	16 (7%)
Vitamin B12 and folic acid	7 (4%)	24 (10%)
Corticosteroids for systemic use, plain	24 (15%)	41 (17%)
Tetracyclines, beta-lactam antibacterials, penicillins	36 (23%)	68 (28%)
Antiinflammatory and antirheumatic products, non-steroids	35 (22%)	92 (38%)
Opioids	18 (12%)	27 (11%)
Other analgesics and antipyretics	20 (13%)	123 (50%)
Antiepileptics	12 (8%)	18 (7%)
Anxiolytics	19 (12%)	30 (12%)
Hypnotics and sedatives	34 (22%)	49 (20%)
Antidepressants	19 (12%)	53 (22%)

Online Resource 3. Baseline characteristics of patients initiating DMF – healthcare resource utilization in the past year

	Treatment-naïve DMF patients N = 156	Patients switching to DMF from other DMTs N = 244
Among all patients		
number of hospitalizations, mean (SD)	0.7 (0.9)	0.4 (0.9)
in-hospital days, mean (SD)	3.9 (9.0)	2.0 (7.8)
Patients with at least one hospital admission, N (%)	77 (49%)	52 (21%)
Among patients with at least one hospital admission		
number of hospitalizations, mean (SD)	1.4 (0.8)	1.7 (1.4)
in-hospital days, mean (SD)	8.0 (12.0)	9.3 (14.8)
Number of outpatient specialist visits, mean (SD)	5.8 (4.0)	5.4 (4.1)
Number of primary care visits, mean (SD)	2.3 (2.7)	2.2 (2.5)
Number of medication classes dispensed, mean (SD)	3.4 (3.2)	6.0 (4.4)
