# Supplementary material for: Persistence with dimethyl fumarate in relapsing-remitting multiple sclerosis: a population-based cohort study
Source: Eur J Clin Pharmacol. 2017 Nov 11;74(2):219–26. doi: 10.1007/s00228-017-2366-4 (PMC5765201; doi:10.1007/s00228-017-2366-4)
Supplement: Supplementary file 4 — (PDF 18 kb) [file 228_2017_2366_MOESM4_ESM.pdf]

Online Resource 4. Switching patterns during the follow-up period (first switching only)

	Switched to							
	**Interferon-betas**	**Glatiramer acetate**	**Fingolimod**	**Teriflunomide**	**Natalizumab**	**Alemtuzumab**	**Rituximab**	**Total**
One year follow-up, N (%)								
Treatment-naïve DMF patients	1 (3%)	2 (6%)	3 (10%)	2 (6%)	1 (3%)	0 (0%)	22 (71%)	31 (100%)
Patients switching to DMF from other DMTs	7 (14%)	3 (6%)	3 (6%)	4 (8%)	6 (12%)	0 (0%)	26(53%)	49 (100%)
Two year follow-up, N (%)								
Treatment-naïve DMF patients	2 (4%)	2 (4%)	4 (8%)	3 (6%)	1 (2%)	0 (0%)	39 (76%)	51 (100%)
Patients switching to DMF from other DMTs	9 (11%)	3 (4%)	4 (5%)	7 (8%)	9 (11%)	1 (1%)	50 (60%)	83 (100%)
All follow-up, N (%)								
Treatment-naïve DMF patients	2 (3%)	2 (3%)	5 (9%)	3 (5%)	1 (2%)	0 (0%)	45 (78%)	58 (100%)
Patients switching to DMF from other DMTs	9 (9%)	3 (3%)	4 (4%)	9 (9%)	10 (11%)	1 (1%)	59 (62%)	95 (100%)
