# Supplementary figures and images for: Persistence with dimethyl fumarate in relapsing-remitting multiple sclerosis: a population-based cohort study
Source: Eur J Clin Pharmacol. 2017 Nov 11;74(2):219–26. doi: 10.1007/s00228-017-2366-4 (PMC5765201; doi:10.1007/s00228-017-2366-4)

**Online Resource 5. Persistence with DMF in all patients initiating DMF**  
**Treatment gap = 60 days**

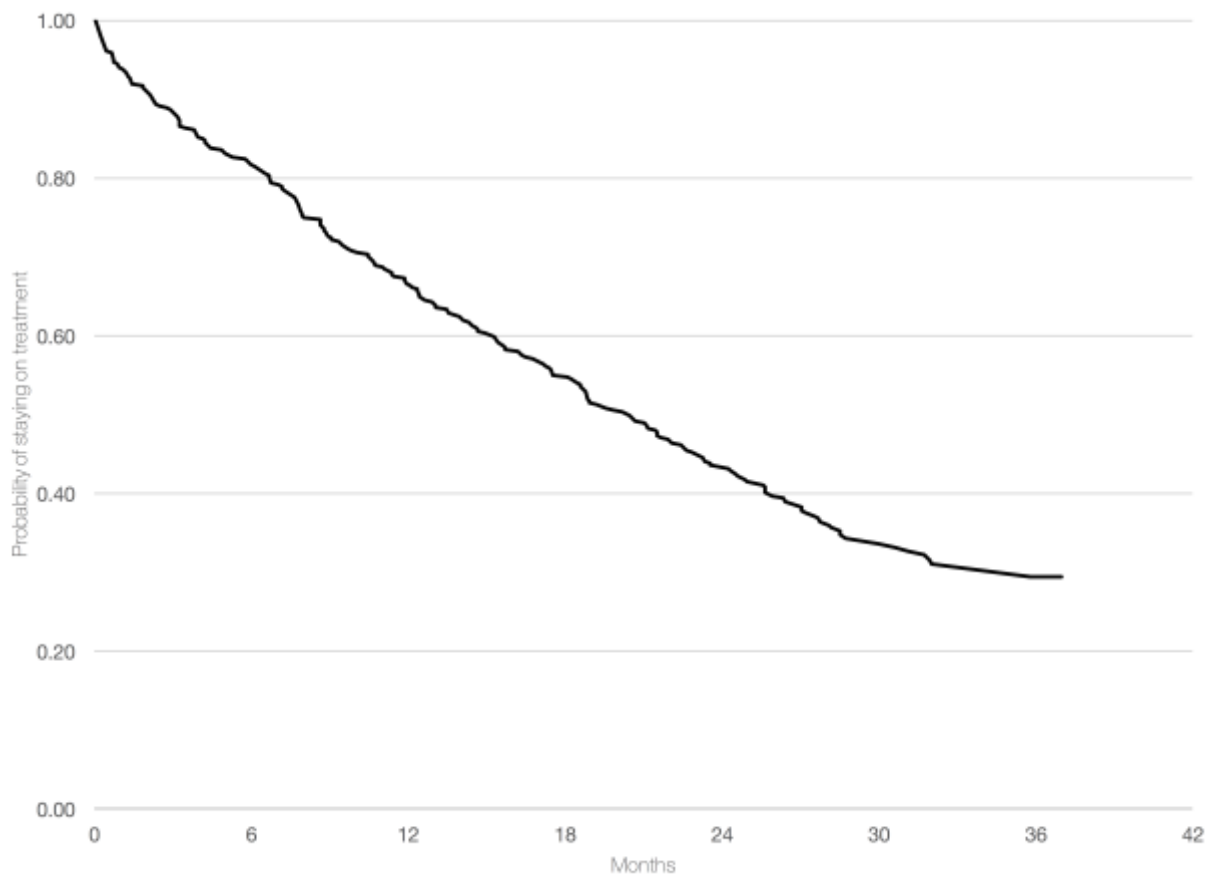

Supplement: Supplementary file 5 — (PDF 20 kb) [file 228_2017_2366_MOESM5_ESM.pdf]
