# Supplementary material for: Persistence with dimethyl fumarate in relapsing-remitting multiple sclerosis: a population-based cohort study
Source: Eur J Clin Pharmacol. 2017 Nov 11;74(2):219–26. doi: 10.1007/s00228-017-2366-4 (PMC5765201; doi:10.1007/s00228-017-2366-4)

Online Resource 6. Sensitivity analyses

Treatment gap = 30 days
Persistence with DMF in all patients initiating DMF

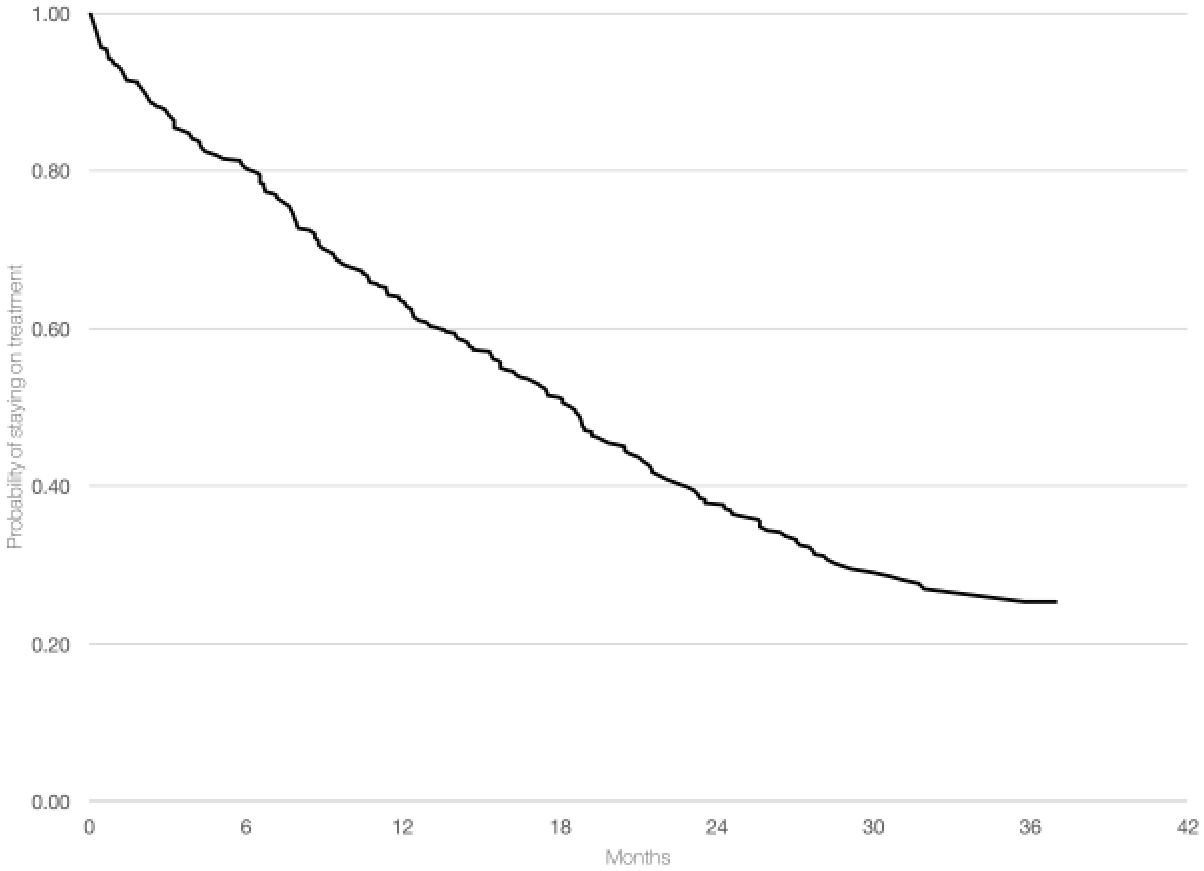

Online Resource 6. Sensitivity analyses

Treatment gap = 30 days
Persistence with DMF in treatment-naïve DMF patients and in patients switching to DMF from other DMTs

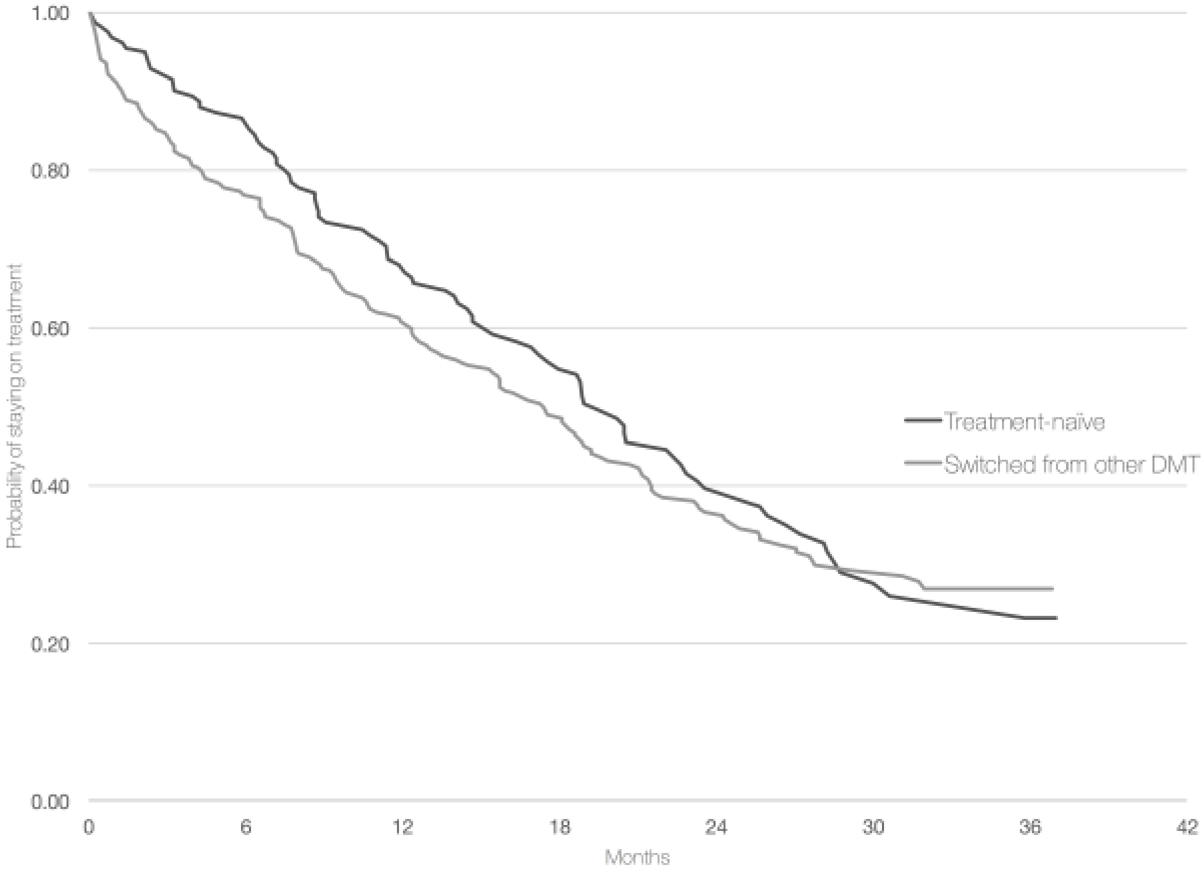

Online Resource 6. Sensitivity analyses

Treatment gap = 180 days
Persistence with DMF in all patients initiating DMF

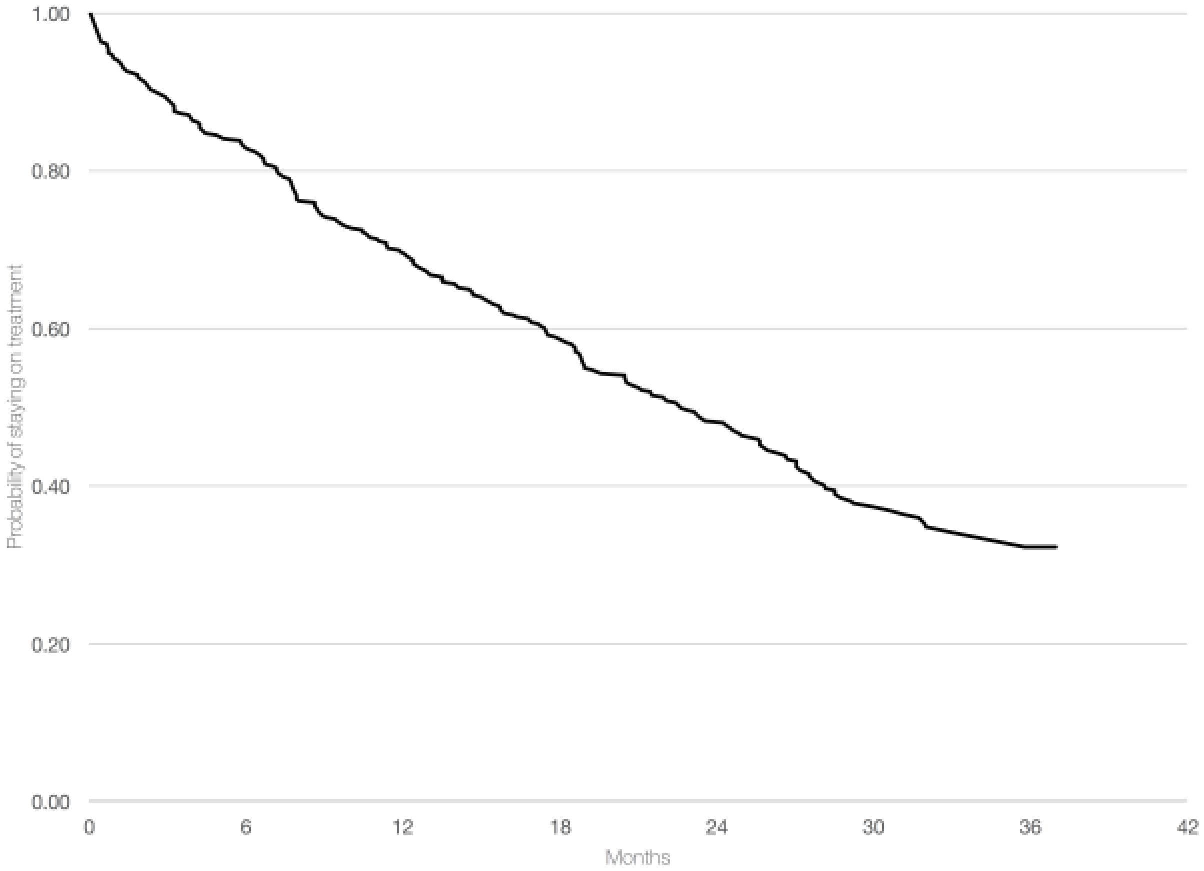

Online Resource 6. Sensitivity analyses

Treatment gap = 180 days
Persistence with DMF in treatment-naïve DMF patients and in patients switching to DMF from other DMTs

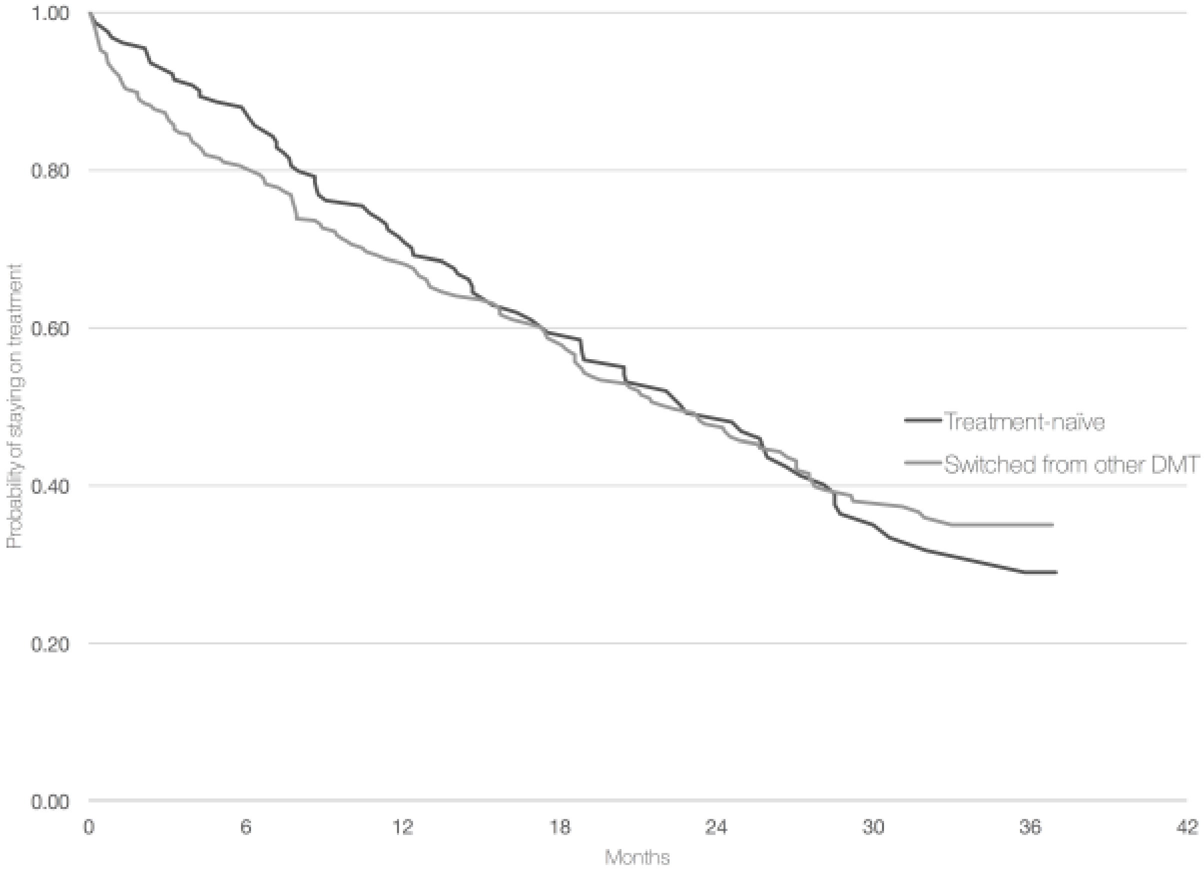

Supplement: Supplementary file 6 — (PDF 77 kb) [file 228_2017_2366_MOESM6_ESM.pdf]
